# Case Report: A rare case of bilateral middle ear tophaceous gout

**DOI:** 10.3389/fsurg.2024.1353116

**Published:** 2024-03-28

**Authors:** Aybige Camurdan, Conrad Riemann, Frank Brasch, Ingo Todt

**Affiliations:** ^1^ Department of Otorhinolaryngology, Head and Neck Surgery, Medical Faculty OWL, Bielefeld University, Campus Klinikum Bielefeld, Bielefeld, Germany; ^2^ Department of Pathology, Medical Faculty OWL, Bielefeld University, Campus Klinikum Bielefeld, Bielefeld, Germany

**Keywords:** computed tomography (CT), hypacusia, hearing loss, gout tophi, gout, inflammation

## Abstract

**Introduction:**

Hypacusia can be caused by various etiologies; however, hearing loss attributed to gouty tophi remains a rare occurrence. This case report presents, for the first time, a bilateral gouty tophi causing hearing impairment.

**Case presentation:**

This report describes a case study involving an 83-year-old Caucasian female patient who presented symptoms of hypacusia, pruritus, and a sensation of pressure in her right ear. A computed tomography scan revealed the presence of non-homogeneous calcified structures in both ears. Following a comprehensive assessment that included pure-tone audiometry and a thorough evaluation of the patient's clinical complaints, a tympanoplasty procedure was initially performed on the right ear. Pathological analysis revealed the presence of gouty tophi. After surgical removal of the tophus, a notable improvement in the patient's hearing threshold was observed. Four months later, a similar surgical intervention was performed on the contralateral ear, achieving a similar positive outcome. The substantial postoperative decrease of bone conduction indicates an inner ear affection by the gout tophi.

**Conclusion:**

Gout tophus in both ears is a very rare but possible cause of hypacusia, even in the absence of a pre-existing diagnosis of systemic gout disease. We report a case of gout tophi in both ears as a rare cause of hearing loss.

## Introduction

Hypacusia is a prevalent symptom with numerous underlying causes (1). These etiologies include a wide range of factors such as embryonic development anomalies, genetic causes of hearing loss, infections, noise exposure, otosclerosis, trauma, ototoxicity, acoustic neuroma, autoimmune diseases, central auditory processing disorder, presbycusis, and Meniere’s disease (2).

Gout is a prevalent metabolic disorder affecting approximately 1%–2% of adults in developed countries, with a higher prevalence observed among middle-aged men and postmenopausal women (3). Gout is a metabolic condition marked by elevated uric acid levels in the body and the accumulation of monosodium urate (MSU) crystals in joints and soft tissues (4). This condition involves the accumulation of solid MSU crystal clusters in various tissues, such as joints, bursae, and tendons. Tophi, which are nodular deposits of these crystals, can develop in various places, including the helix of the ears, the olecranon bursa, and around the interphalangeal joints (4).

In the literature, there is a limited publication on cases of gout affecting the ear, with reports focusing only on unilateral involvement.

To the best of our knowledge up to the year 2022, only 10 cases of gout localized in the head and neck region have been documented. These cases include occurrences such as subcutaneous nodules on the helix and deposits in the larynx, nasal septum, and the middle ear (5). In patients who might otherwise not show any clinical or biochemical indications of gout, it is frequently misinterpreted as cholesteatoma or tympanosclerosis. Despite its rarity, gout can cause serious illnesses, including conductive hearing loss (6).

We present a case of a patient who experienced right-sided deafness and left-sided conductive hearing loss caused by gout tophi. Remarkably, the patient underwent bilateral tympanoplasty procedures within four months, resulting in an improvement of conductive and even sensorineural hearing.

## Case presentation

**Figure 1:**
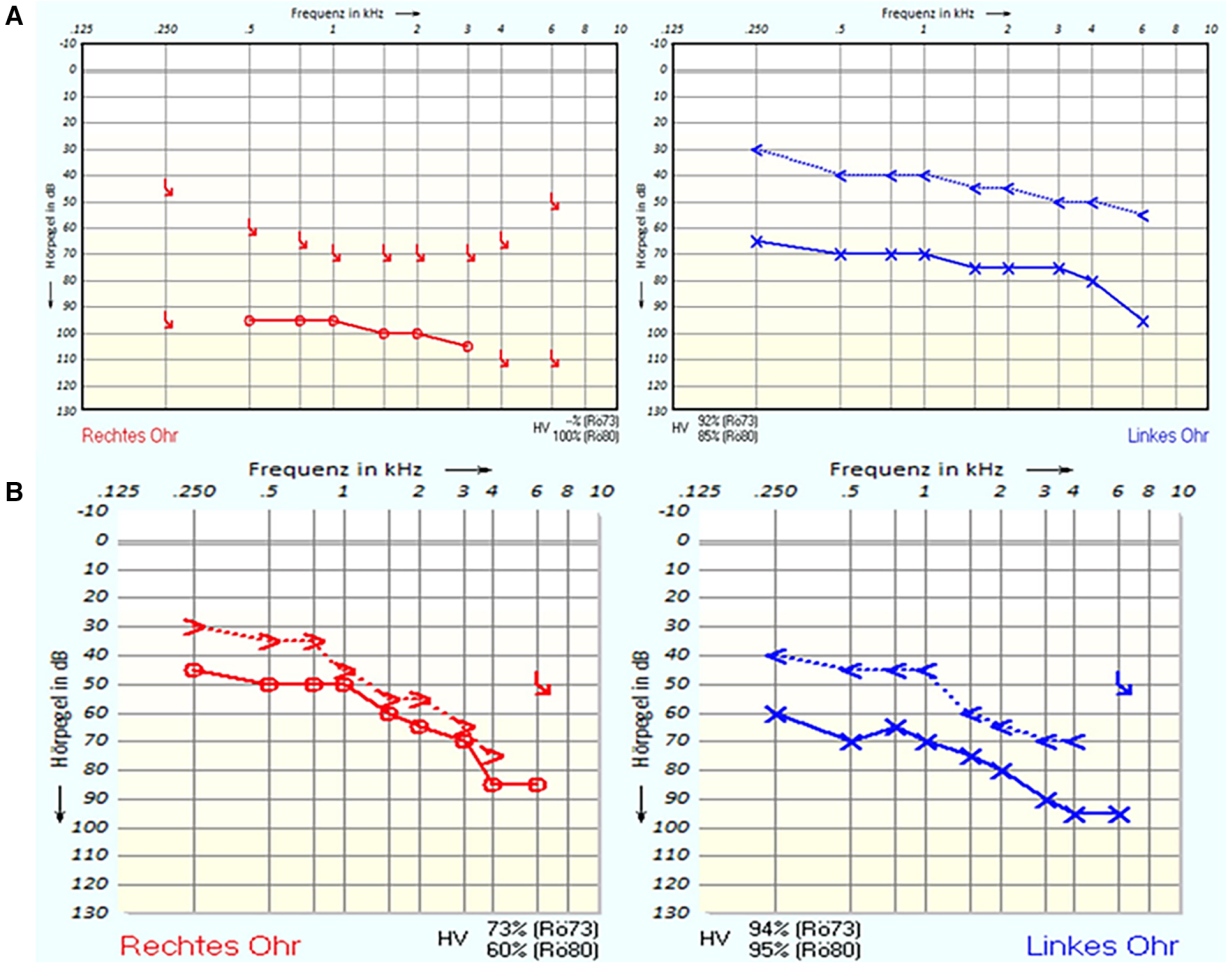
(**A**) Pure-tone audiometry (PTA) before the first surgery. (**B**) PTA after the second surgery.

In this report, we present a case study of an 83-year-old Caucasian female patient who presented with symptoms of hypacusia, predominantly affecting the right ear, accompanied by sensations of itchiness and perception of pressure within the same ear. The initial otoscopy examination revealed the presence of an inhomogeneous white, shiny mass. In the first evaluation, an osteoma was considered as a differential diagnosis for cholesteatoma. The pure-tone audiometry (PTA) revealed deafness on the right side and a 30 dB air–bone gap indicating mixed hearing loss on the left side (Figure 1A). Repeated measurements confirmed the hearing threshold.

The patient suffered from known chronic diseases such as hypothyroidism, polyneuropathy, diabetes mellitus type II, high blood pressure, and right bundle branch block. Her medications include L-thyrox, valsartan, metformin, torasemide, doxazosin, Turfa, Betmiga, atorvastatin, pantoprazole, painkiller, aspirin, and insulin. At the time of evaluation, she did not describe any gout symptoms.

**Figure 2:**
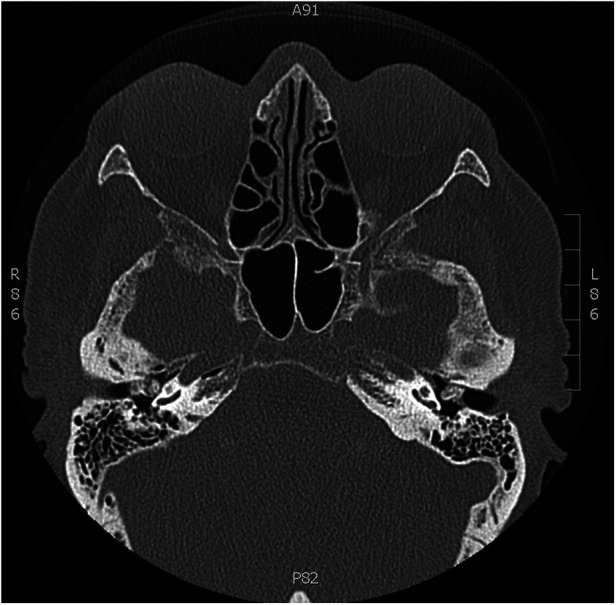
CT scan of temporal bones. Bilateral irregular calcified lesion with regular borders that cannot be distinguished from the eardrum and does not damage the ossicular chain.

High-resolution computed tomography of the temporal bone showed in both middle ears irregularly calcified masses measuring approximately 7.6 mm by 6 mm on the right and 6 mm by 5 mm on the left. These masses have smooth edges and are situated broadly and predominantly toward the front of the auditory canal, without any detectable damage to the surrounding bone. They closely adjoin the eardrum and cannot be separated from it. The eardrum appears slightly thickened, with no sign of fluid accumulation. In conclusion, these findings suggest benign changes, possibly osteoma. Additionally, proper ventilation of the mastoid cell system was observed on both sides (Figure 2).

**Figure 3:**
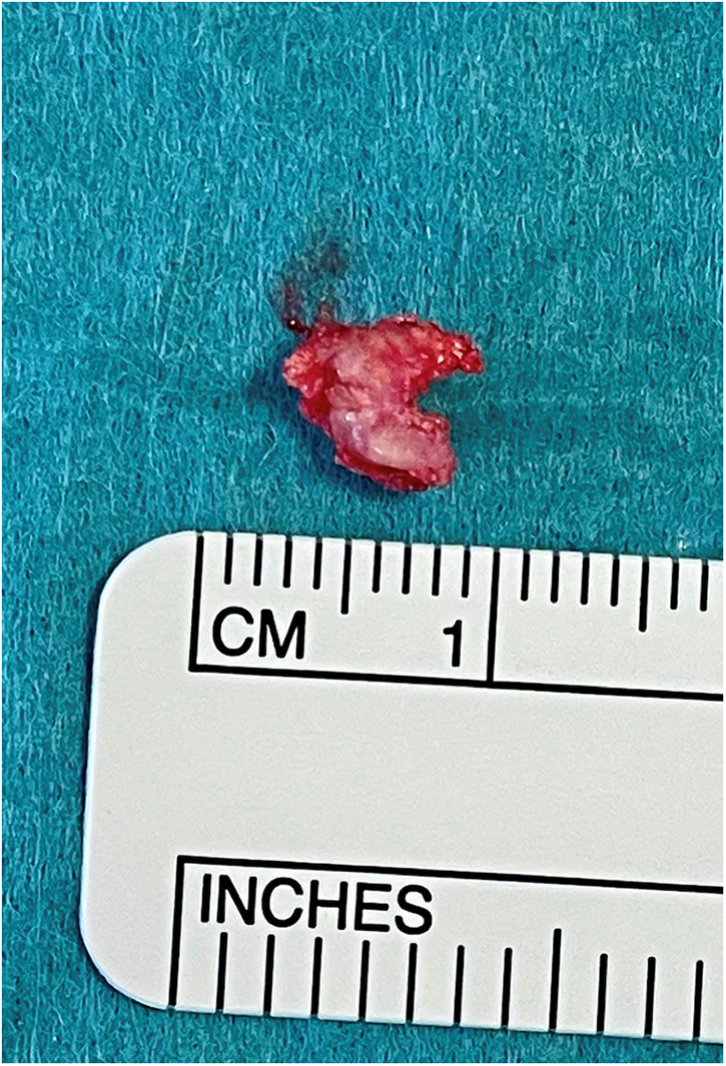
Intraoperative finding after removing the mass from the tympanic membrane.

Based on these findings, the patient underwent an initial right tympanoplasty to remove the calcified mass (Figure 3). The tumor was attached to the tympanic membrane that was reconstructed by thin cartilage. The ossicular chain was intact but surrounded by the mass. A reconstruction of the chain was not necessary. The mass was even partially lying in the oval niche.

**Figure 4:**
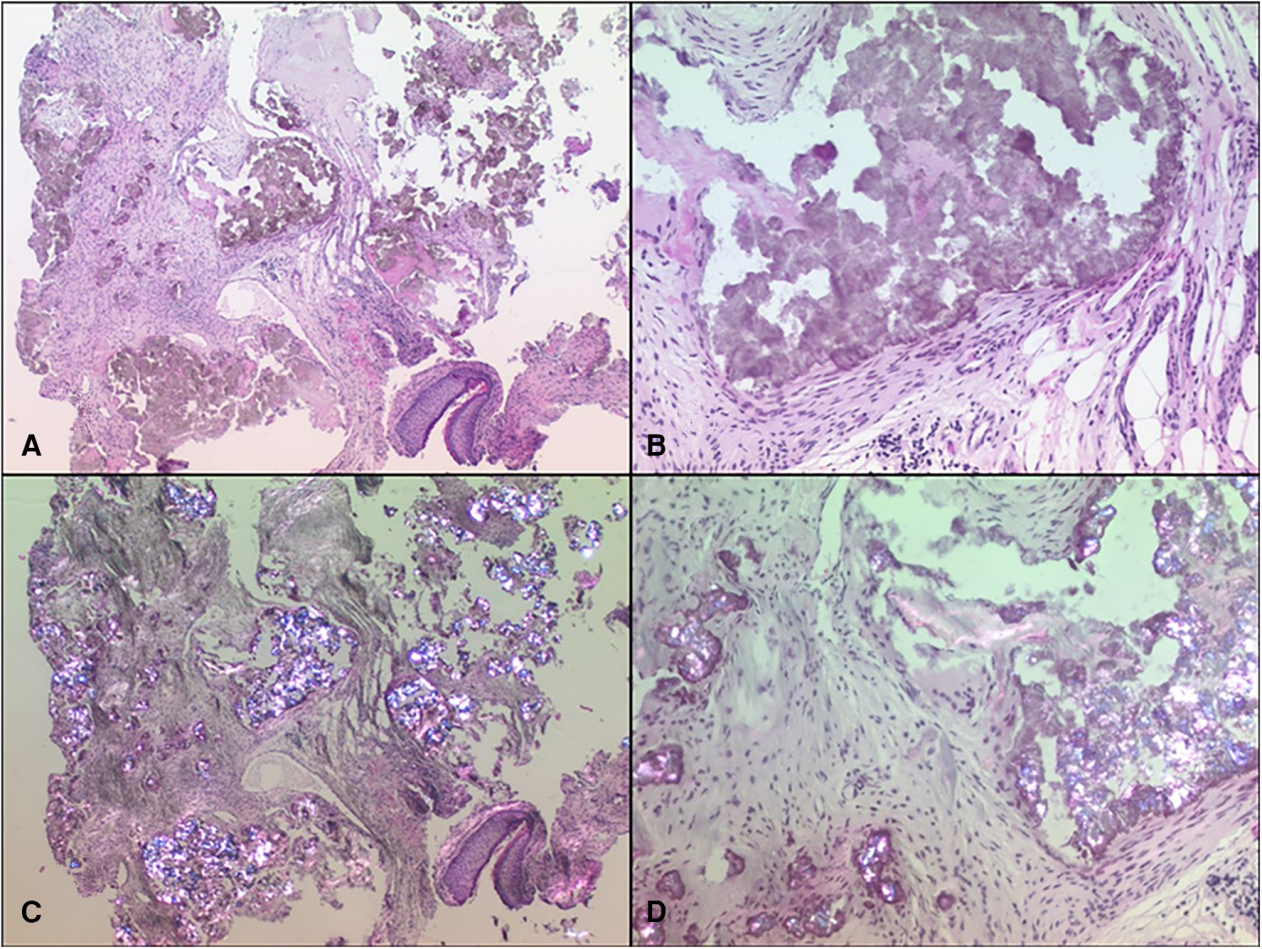
Two samples at both 25× (**A**,**C**) and 100× (**B**,**D**) magnifications: One using standard hematoxylin and eosin (HE) staining in normal light microscopy (**A**,**B**) and the other in polarization microscopy (**C**,**D**), where urate crystals exhibited a distinct brilliance.

The histopathological examination showed fibroid stroma from the tympanic membrane, containing extensive deposits of a dirty brownish material that exhibited optical polarization partial radiance, and clumpy birefringence. These findings are primarily indicative of a gouty tophus, ruling out the possibility of pseudogout or chondrocalcinosis (Figure 4).

Four months after the first right-sided surgery, on the left ear, a tympanoplasty was performed. Histopathological analysis confirmed that the lesion in the left ear was consistent with a gouty tophus, like the lesion in the right ear. After the removal of ear tamponade on both sides, the hearing threshold in PTA improved (Figure 1B). In addition, after the histological detection of gout tophi, in the blood sample, there was no evidence of increased uric acid level. An auditory rehabilitation with hearing aids was denied by the patient.

## Discussion

Gouty tophi within the middle ear is exceptionally uncommon and serves as a rare differential diagnosis for middle ear masses, often contributing to conductive-type hearing loss (7). Because of that, based on clinical and radiological findings, cholesteatoma, osteoma, or tympanosclerosis is generally considered the first diagnosis (6). Most of the reported case studies on middle ear gouty tophi indicate a favorable surgical prognosis, as they can often be completely removed without affecting the integrity of the ossicular chain (6).

The case report highlights that bilateral gouty tophi can lead to sensorineural hearing loss. Additionally, a pure tympanoplasty with the removal of the tophi affects the inner ear function. The underlying mechanism of how this local gout focus affects the inner ear function remains speculative. Local transmission of ureate into the inner ear can be proposed since parts of the tophi were located in the oval nice. Gout itself is a known risk factor for the development of inner ear hearing loss based on its inflammatory properties (8). Usually, surgical removal of the mass leaves an intact regularly moving ossicular chain (5, 6).

Distinguishing a middle ear mass solely based on medical history, clinical examination, and radiological assessments can be challenging. Surgical intervention and subsequent histopathological examination remain the definitive and most reliable diagnostic approach. Furthermore, complete removal of the mass during surgery offers a curative treatment option.

## Conclusion

Hypacusia may be caused by gouty tophus. Performing a tympanoplasty can detect and cure this uncommon middle ear pathology.

## Data Availability

The original contributions presented in the study are included in the article/Supplementary Material, and further inquiries can be directed to the corresponding author.
